# Pediatric Traumatic Cervical Distraction Injury: A Case Report

**DOI:** 10.7759/cureus.62910

**Published:** 2024-06-22

**Authors:** Seiya Watanabe, Kazuo Nakanishi, Kazuya Uchino, Hideaki Iba, Yoshihisa Sugimoto, Shigeru Mitani

**Affiliations:** 1 Orthopedics, Kawasaki Medical School, Okayama, JPN

**Keywords:** infant, surgical operation, spinal cord injury, pediatric cervical spine, pediatric trauma

## Abstract

Spinal cord injury due to trauma is rare in children. We report our experience with the surgical treatment of a cervical spinal cord injury in a one-year-old child with quadriplegia due to traffic trauma. The patient was a girl aged one year and five months. Physical examination findings were quadriplegia and loss of consciousness. Plain computed tomography (CT) of the cervical spine showed a vertical distraction injury of C6/7, and magnetic resonance imaging (MRI) showed spinal cord injuries of C1/2 and C6/7. Based on these findings, a diagnosis of C1/2 and C6/7 spinal cord injury (Frankel A) was made. The patient’s state of consciousness did not change during the first week after injury; she was managed systemically with a ventilator. On the 10th day after the injury, her consciousness improved, and she was placed in a pediatric halo vest for weaning. However, as the alignment worsened, we operated. A 5 cm posterior incision was made at the median of C5/6/7. Only the spinous process was deployed, a Nespron tape (Alfresa Pharma Corporation, Osaka, Japan) was wrapped between C5/6 and C6/7, and an autologous iliac bone graft was placed at the C6/7 bilateral facet joint. Six months after surgery, bone fusion was complete. At one year and six months postoperatively, tetraplegia had not improved. Radiographs showed no growth disturbances despite residual alignment abnormalities.

## Introduction

Cervical spine injuries in children are rare. It is about 1 to 2% of all pediatric injuries [1,2]. The mean age of injury for spinal cord injury in children is 13.0 ± 4.0 years [3]. There are few reports of surgery for spinal cord injury in infants. Therefore, it is difficult to determine a treatment plan for infants who require surgery. We report our experience with the surgical treatment of a cervical distraction injury in a one-year-old child with quadriplegia due to traffic trauma.

## Case presentation

**Figure 1:**
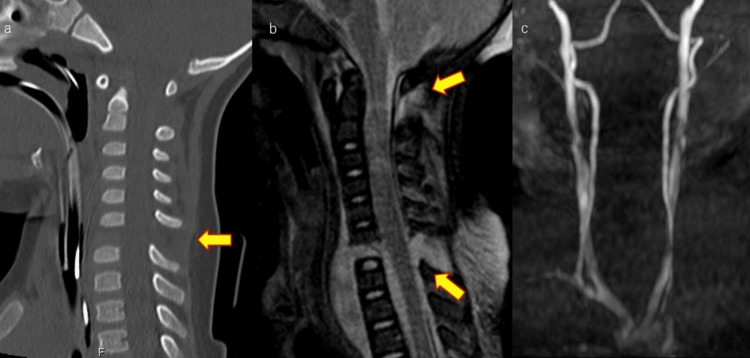
Preoperative CT and MRI and MRA. (a) Sagittal CT of the cervical spine (preoperative). Arrows indicate C6/7 distraction injury. (b) Sagittal MRI of the cervical spine (preoperative). Arrows indicate C1/2 and C6/7 cervical cord injury. (c) MRA of the cervical spine (preoperative). MRA shows no vertebral artery damage. CT: Computed tomography, MRI: Magnetic resonance imaging, MRA: Magnetic resonance angiography.

The patient was a girl aged one year and five months. She was brought to our hospital by helicopter after being hit by a truck while riding in a car. She was sitting forward facing in the passenger seat with a child car seat. On arrival, she was 79 cm tall, weighed 9.5 kg, and had a Glasgow Coma Scale (GCS) of E1(eye response: none)/V1(verbal response: none)/M1(motor response: none), with no spontaneous respiration or limb movements. The GCS, reported by Teasdale in 1976, is a method of assessing impaired consciousness by eye-opening, verbal, and motor response [4]. Physical examination findings were quadriplegia and loss of consciousness. Plain computed tomography (CT) of the cervical spine showed a vertical dislocation of C6/7, magnetic resonance imaging (MRI) showed spinal cord injuries of C1/2 and C6/7, and MRA (magnetic resonance angiography) showed no vertebral artery damage (Figure 1).

**Figure 2:**
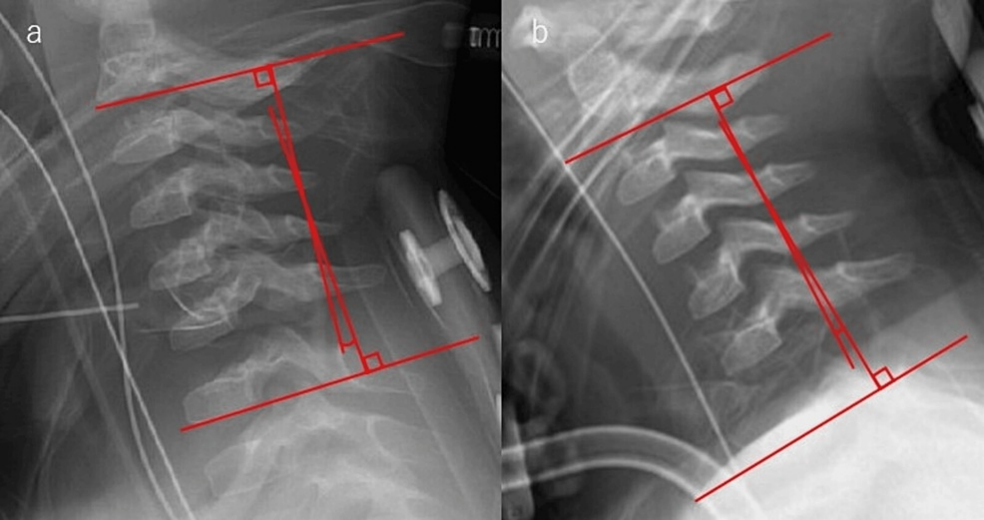
X-ray (preoperative). (a) X-ray immediately after fixation (cervical lordosis angle is 8°). (b) X-ray one week after fixation (cervical lordosis angle is 2°). We consider that the cervical lordosis angle is decreased, and the alignment is worsened.

Based on these findings, a diagnosis of C1/2 and C6/7 spinal cord injury (Frankel A) was made. The patient’s state of consciousness did not change during the first week after injury; she was managed systemically with a ventilator. On the 10th day after the injury, her consciousness improved, and she was placed in a pediatric halo vest for weaning. However, as the alignment worsened, we operated (Figure 2). 

**Figure 3:**
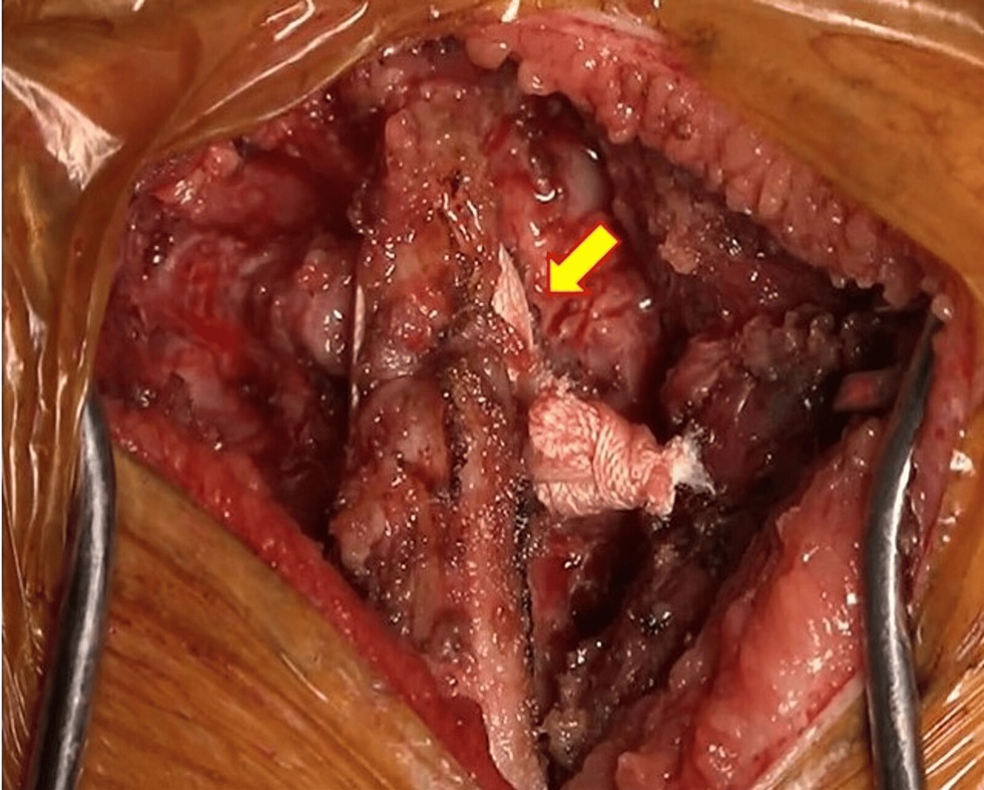
Intraoperative findings. The arrow indicates the Nespron tape wrapping around the spinous process.

A 5 cm posterior incision was made at the median of C5/6/7. Only the spinous process was deployed, a Nespron tape (polystyrene thread) made by Alfresa Pharma Corporation (Osaka, Japan) was wrapped between C5/6 and C6/7, and an autologous iliac bone graft was placed at the C6/7 bilateral facet joint (Figure 3).

**Figure 4:**
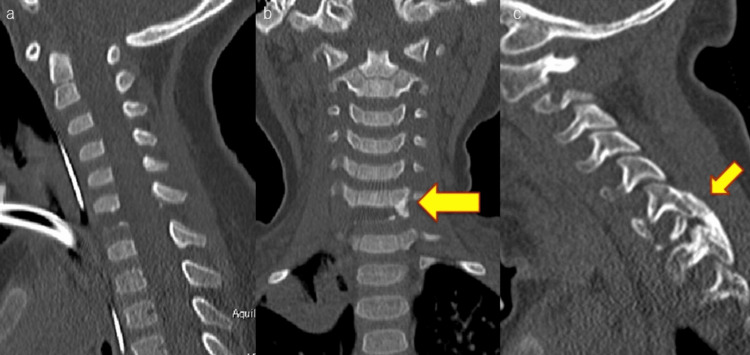
Postoperative CT (a) Sagittal CT of the cervical spine (one month postoperatively). (b) Coronal CT of the cervical spine (one month postoperatively). (c) Simple CT of the cervical spine (six months postoperatively). The arrows indicate the callus. Bone fusion is progressing.

The operative time was 76 min and blood loss was minimal. Postoperatively, the patient was again fitted with a halo vest. One month postoperatively, a plain CT scan of the cervical spine showed bone fusion, and the halo vest was removed. Six months after surgery, bone fusion was complete (Figure 4).

**Figure 5:**
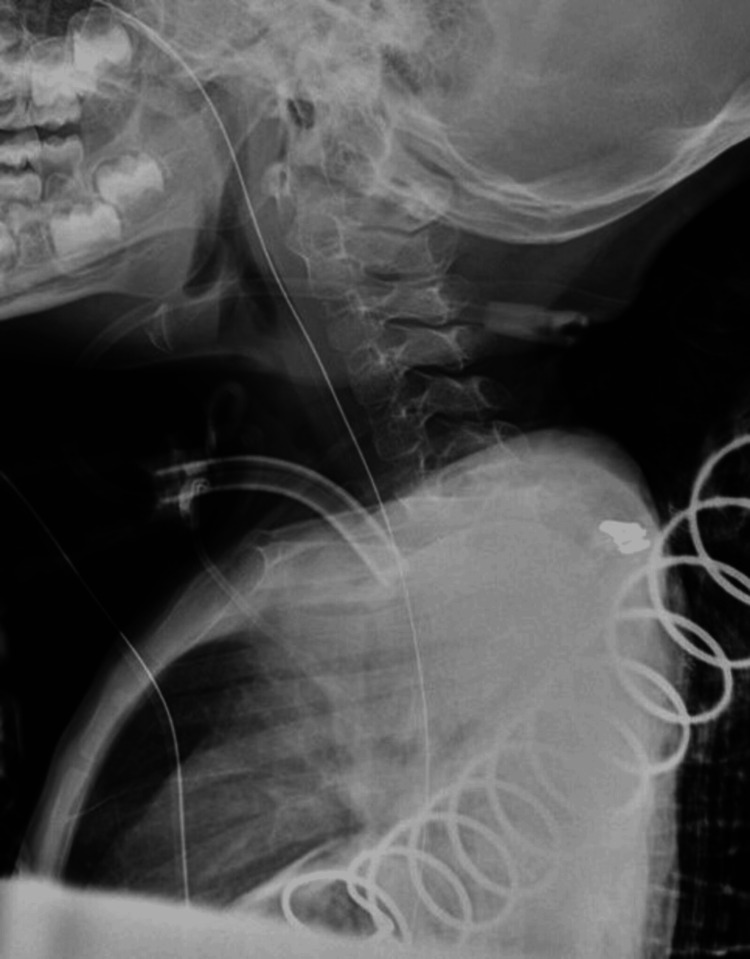
Cervical spine X-ray (one year and six months postoperatively)

The patient was then transferred to another hospital for recuperation. At one year and six months postoperatively, tetraplegia had not improved. Radiographs showed no growth disturbances despite residual alignment abnormalities (Figure 5). It was difficult for the patient to go to our hospital. From now on, they were admitted to a children's care facility and sent for X-rays every six months and referred if there were abnormalities.

## Discussion

Pediatric cervical cord injuries are rare, accounting for approximately 1 to 2% of all pediatric traumatic injuries [1,2]. These injuries are difficult to diagnose and treat because there is no consensus on the diagnosis and treatment of pediatric cervical spinal cord injuries. Spinal cord injury in children often occurs without evidence of fracture or dislocation. Pang et al. reported spinal cord injuries without fractures or dislocations as SCIWORA (spinal cord injury without radiographic abnormality) [5]. Several anatomical characteristics of the pediatric spine increase its susceptibility to spinal cord injury. Children have a different cervical fulcrum compared to adults due to their disproportionately large heads. In children, the ligaments, posterior joint capsules, and cartilaginous structures are more elastic than the rigid adult spine and, therefore, more deformable. The planes of the facet joints in children are also more horizontal and thus possess greater mobility but less stability [5,6]. Prevention is important because spinal cord injuries are difficult to treat once they occur. The most common cause of spinal cord injury is traffic crashes [7]. This case was also injured by a traffic crash. She was sitting forward-facing in the passenger seat with a child car seat. Proper child car seat use should be rear-facing in the back seat [8]. Facing the rear minimizes the risk of head and neck injury in the event of a crash. If an infant is facing forward, the harness restrains the body, but the head and neck remain unrestrained and whip forward in rapid flexion, potentially causing injury [9].

If pediatric cervical spinal cord injury has occurred, conservative therapy is the primary treatment [10]. However, external fixation such as a halo vest is difficult because of the elasticity of the cervical spine in children [11]. In addition, the skull of children is thinner than that of adults, and the fixation of pins is weak, making treatment difficult due to loosening of pins, infection, or perforation of screws [12]. In the present case, a halo vest was used; however, malalignment occurred. There are only a few reports of surgical treatment of cervical spinal cord injuries in children. Li et al. reported a three-year-old child with cervical spinal cord injury who underwent anterior cervical fusion (C6/7) and posterior fusion with cervical pedicle screws (C5-T1) [13]. The patient underwent posterior cervical spine fusion using a pedicle screw (PS) with a 3.5 mm diameter screw. The diameter of the pedicle in children is 3-4 mm [14]. However, in our case, the one-year-old child had a pedicle diameter of 1.5 mm as measured on preoperative CT was approximately 1.5 mm, which made it difficult to insert a PS. Moreover, the patient had a C1/2 cervical spinal cord injury that resulted in respiratory compromise and required ventilator management via tracheotomy. We thought an anterior autologous bone graft was necessary. However, the location of the tracheotomy and the anterior incision were too close together. Therefore, we could not perform an anterior fixation. The patient was treated with Nespron tape (Alfresa Pharma Corporation, Osaka, Japan), an autologous bone graft (iliac bone), and a postoperative halo vest to achieve bone fusion with minimal growth disturbance. Tetraplegia had not improved one year and six months after surgery. Although there were alignment abnormalities on radiography, there were no growth disturbances, and the treatment results were judged to be relatively good.

A limitation of this study is the short follow-up period. It is possible that the deformity will progress as the patient grows in the future. Therefore, follow-up should be conducted over a long period of time.

## Conclusions

We reported a spinal cord injury in a one-year-old child. Spinal cord injuries in infants are rare. It is important that the treatment of children does not cause growth disturbances. We used Nespron tape (Alfresa Pharma Corporation, Osaka, Japan), autologous iliac bone graft, and postoperative halo vests to avoid growth disturbances as much as possible. At one year and six months postoperatively, there is no improvement in paralysis but no growth disturbance. It is important to prevent spinal cord injuries by using a proper child car seat.
